# Correlation Entropy and Power-Law Kinetics

**DOI:** 10.3390/e28060712

**Published:** 2026-06-21

**Authors:** Joseph B. Bernstein

**Affiliations:** Department of Electrical and Electronics Engineering, Ariel University, Ariel 40700, Israel; josephbe@ariel.ac.il

**Keywords:** correlation constant, power-law, kinetics, statistical mechanics, Gibbs free energy, thermodynamics, correlated systems

## Abstract

Power-law kinetics are observed across a wide range of physical, chemical, biological, and engineering systems, yet the thermodynamic origin of the power-law exponent remains incompletely understood. This work proposes a thermodynamic hypothesis in which power-law behavior emerges naturally from correlation-dependent contributions to the Gibbs free energy. Rather than modifying the classical Boltzmann definition of entropy, a phenomenological Correlation Constant, χ, is introduced to quantify how accumulated microstate evolution influences the accessibility of future states. The resulting correlation entropy contribution produces a free-energy term that modifies the probability of subsequent transitions and leads naturally to power-law kinetic behavior. Positive values of χ correspond to cooperative evolution in which prior evolution promotes future evolution, while negative values correspond to self-limiting behavior in which prior evolution suppresses subsequent evolution. The conventional Arrhenius-Eyring description is recovered as the special case χ = 0. The resulting framework provides a thermodynamic interpretation of the power-law exponent, establishes a connection between entropy, free energy, and kinetic evolution, and offers a unified description applicable to degradation, relaxation, diffusion, fatigue, trapping, and other evolving processes. The present work is intended as a thermodynamic hypothesis motivating further experimental and theoretical investigation of correlation-dependent kinetics.

## 1. Introduction

The scientific method begins with observation. Experimental phenomena are identified, patterns emerge, hypotheses are proposed, and physical theories are subsequently developed to explain the observations. Throughout the history of science, many of the most successful theories have followed this progression. Empirical relationships often appear long before a complete physical understanding is achieved, and the eventual success of a theory is determined not only by its ability to describe observations, but also by its ability to unify apparently unrelated phenomena within a common framework.

An important challenge in this process is that once a hypothesis has been proposed, there is a natural tendency to interpret subsequent observations in a manner consistent with the prevailing explanation. The methods used to collect, analyze, and present experimental data may therefore influence the conclusions that are ultimately drawn. For this reason, it is occasionally useful to revisit well-established physical descriptions and examine whether alternative interpretations of familiar observations may exist.

One of the most influential examples in physical science is the description of thermally activated processes. Since the pioneering work of Arrhenius, the temperature dependence of reaction rates has provided a remarkably successful framework for understanding phenomena in chemistry, physics, materials science, and engineering. The Arrhenius relationship introduced the concept that a process rate may be governed by an energetic barrier and established a quantitative connection between microscopic energetics and macroscopic behavior [1].

The success of the Arrhenius framework motivated subsequent efforts to place energetically or thermally activated processes on a broader theoretical foundation. Statistical interpretations of thermally activated phenomena emerged from the development of statistical mechanics while later work sought to connect reaction rates directly to thermodynamic quantities. These developments culminated in transition-state theory and the recognition that free energy, rather than energy alone, governs the evolution of many physical systems.

At the same time, advances in condensed matter physics revealed that many systems cannot be fully described as collections of independent events. Disorder, localization, occupancy, and interaction effects play an important role in determining the behavior of materials ranging from metal fatigue and semiconductor devices to disordered systems. The energetic landscape experienced by a system may itself evolve as the system changes, leading to behavior that is not easily described within a fixed-barrier Arrhenius framework.

The purpose of the present hypothesis is to reexamine the relationship between thermodynamics, kinetics, and interactions in evolving physical systems. By tracing the historical development of activated processes from classical reaction-rate theory through modern concepts of disorder and correlation, we seek to establish a new framework capable of describing a broad class of experimentally observed kinetic behavior within a common thermodynamic perspective. Specifically, the focus here is to understand better the power-law temporal evolution behavior observed in various real-world applications.

### 1.1. Historical Development of Activated Processes

The modern understanding of kinetic processes can be traced to the work of Arrhenius in the late nineteenth century [1]. His approach seeks to explain the strong temperature dependence observed in chemical reaction rates. Arrhenius proposed that reactions proceed through a thermally activated state. This reaction rate then depends exponentially on an activation energy. The resulting Arrhenius relationship became one of the most successful empirical laws in science and established the idea that macroscopic rates may be governed by microscopic energetic barriers.

Although the Arrhenius equation successfully described a wide range of experimental observations, it did not provide a fundamental explanation for the origin of the activation process itself. Such an interpretation emerged through the development of statistical mechanics. Einstein’s work on Brownian motion and random processes demonstrated how the collective behavior of large numbers of microscopic events could produce predictable macroscopic laws [2]. These developments established a statistical foundation for activated behavior and reinforced the concept that macroscopic physical evolution may be understood in terms of transitions between microscopic states.

A major advance occurred with the development of transition-state theory by Eyring and coworkers [3,4]. Rather than describing reaction rates solely through an activation energy, Eyring expressed the rate in terms of the Gibbs free energy of activation. In doing so, the theory explicitly introduced both energetic and entropic contributions to the activation process. Eyring noted that many commonly used rate equations could be viewed as “specializations of a general theory” [3], suggesting that apparently different kinetic behaviors may arise from a common thermodynamic foundation.

The transition from Arrhenius kinetics to a Gibbs free-energy formulation represents an important conceptual shift. While the Arrhenius description emphasizes a fixed activation barrier, the Gibbs formulation recognizes that both energy and entropy contribute to the accessibility of an activated state [3,4]. Consequently, the evolution of a system may depend not only on the magnitude of an energetic barrier but also on the number of microscopic configurations available to the system as its entropy evolves.

The introduction of entropy into kinetic theory provided a natural bridge between reaction-rate theory and statistical mechanics. It also opened the possibility that interactions, disorder, and configurational constraints could influence kinetic behavior through their effect on the free-energy landscape. These ideas would later become central to the study of disordered and non-crystalline materials [5,6,7].

Recent work has employed generalized Wiener degradation models, stochastic diffusion coefficients, and random-process formulations to describe nonlinear degradation kinetics and lifetime prediction. While such approaches can successfully represent observed power-law behavior, the origin of the power-law exponent is typically introduced empirically through the model structure rather than derived from an underlying thermodynamic mechanism [8].

### 1.2. Disorder, Occupancy, and Correlation

The development of transition-state theory established the importance of free energy in determining kinetic behavior. However, many physical systems cannot be adequately described as collections of independent events evolving within a fixed energetic landscape. Beginning in the middle of the twentieth century, increasing attention was directed toward the role of disorder, localization, occupancy, and interaction effects in determining the behavior of complex materials [5,6,9].

A particularly important contribution was made by Mott in his investigations of non-crystalline materials and amorphous semiconductors [5,6]. In contrast to ideal crystalline systems, where electronic states are often described by well-defined energy bands, disordered materials exhibit localized states, trapping phenomena, and a broad distribution of localized traps. Mott emphasized that non-crystalline systems contain a “continuous range of such localized states” [6], providing a physical basis for occupancy-dependent transport through hopping between localized charge states.

The concept of localized states represented an important departure from the assumption that all microscopic transitions occur within a fixed and uniform environment. As localized states become occupied, the energetic landscape experienced by subsequent carriers becomes altered through changes in local fields with screening, relaxation, or other interaction mechanisms. Consequently, the accessibility of available states may evolve as the system becomes progressively charged or discharged.

Related ideas emerged in the study of amorphous and disordered electronic materials. Adler and coworkers emphasized that the behavior of such systems could not completely be understood solely in terms of simple thermal activation, but required consideration of electronic structure, interactions, and correlation effects [7]. These studies suggested that the energetic barriers governing microscopic transitions may in fact depend on the occupancy and state of the surrounding system rather than remaining constant throughout the evolutionary trapping and de-trapping processes.

From a thermodynamic perspective, these observations raised an important question that remains relevant today. If the number of accessible configurations changes as a system composed of an ensemble of microstates evolves, then the entropy contribution to the Gibbs free energy should also evolve. In such cases, the effective free-energy landscape governing subsequent transitions need not remain fixed. If so, the charge trap and microstate evolution of the system would influence the probability of future transitions, producing either cooperative behavior, independent behavior, or self-limiting behavior depending on the nature of the interactions.

The concepts of disorder, occupancy, and correlation therefore provide a natural connection between statistical mechanics and kinetic charge or defect evolution. They suggest that the thermodynamic description of an evolving system may require consideration not only of energetic barriers, but also of the changing configurational accessibility of microscopic states. This observation forms the basis for the discussion that follows.

### 1.3. Motivation for the Present Work

The concepts of activation entropy, disorder, occupancy, and correlation energies suggest that the free-energy landscape governing an evolving system need not remain fixed. If the accessibility of microscopic states (microstates) changes during the evolution process, then the entropy contribution to the Gibbs free energy would necessarily also change. Consequently, the probability of future transitions may depend on the current state of the system itself.

Evidence for such behavior appears throughout physics, chemistry, materials science, and reliability engineering. A wide variety of experimentally observed phenomena exhibit power-law evolution of the form

(1) $$\Delta P=At^{n},$$

where ΔP represents an accumulated change in a measurable parameter and *n* is a kinetic exponent of time, *t*. Although the underlying physical mechanisms may be substantially different, similar mathematical forms have been reported for fatigue, degradation, trapping, transport, relaxation, and other irreversible processes [10,11,12,13].

From a phenomenological perspective, three broad classes of kinetic behavior are commonly observed:

- In cooperative systems, the probability of future evolution increases as damage accumulates, leading to accelerating behavior characterized by n>1.
- In independent systems, the probability of future evolution remains unchanged, resulting in linear behavior with n=1.
- In self-limiting systems, the probability of future charge or defect evolution decreases as the system evolves, producing retarding behavior characterized by 0<n<1.

Although these regimes are frequently treated as distinct classes of phenomena, they may represent different manifestations of a common underlying thermodynamic process.

The central question addressed in this work is whether these apparently different kinetic behaviors can be described within a unified thermodynamic framework. Here, we are motivated by the historical development of activated processes and by the role of disorder, occupancy, and interaction effects in evolving systems. The present work should therefore be viewed as a thermodynamic hypothesis that the kinetic exponent may reflect a correlation-dependent contribution to the entropy of a system or ensemble of microstates.

To explore this hypothesis, a correlation constant is introduced to characterize the influence of a system’s state on the subsequent evolution of future states. It will be shown that a correlation-dependent entropy contribution to the Gibbs free energy naturally produces cooperative, independent, and self-limiting kinetic behavior within a single mathematical framework. Importantly, the familiar Arrhenius description emerges as a special limiting case corresponding to the absence of correlation effects with n=1. Furthermore, we address the possibility that there can be a single framework that accounts for different kinetics, including the full range including n>1 and 0<n<1.

Before developing the thermodynamic formulation, it is useful to examine some experimental observations that motivate this framework.

## 2. Experimental Motivation

Before developing a thermodynamic description of the kinetics, it is useful to examine a common empirical observation that appears across a broad range of physical systems. Consider an observable system property, *P*, evolving under an applied stress, Ψ, at temperature, *T*. The stress, Ψ, may represent voltage, electric field, current density, mechanical strain, vibration amplitude, radiation flux, chemical concentration, or any other external driving force capable of producing irreversible evolution in defects, charges, strain or others. In many electronic systems, the accumulated change, ΔP, or threshold voltage change, $\Delta V_{th},$ that would be the observable parameter, *P*, that may be represented empirically as

(2) $$\Delta P\left(\Psi ,T,t\right)=A\left(\Psi ,T\right)t^{n\left(\Psi ,T\right)},$$

where $A\left(\Psi ,T\right)$ is a stress and temperature dependent prefactor and $n\left(\Psi ,T\right)$ is the observed kinetic exponent. Although this relationship is frequently treated as purely empirical, power-law behavior has been reported in a remarkably diverse collection of physical systems [10,11,12,13,14].

Of particular interest is the observation that the exponent itself is often found to vary with stress and temperature. Such behavior suggests that the exponent may contain physical information regarding the evolving state of the system due to applied stress and temperature, Ψ and *T*. This contrasts with a more standard assumption that *n* serves only as an un-physical fitting parameter. The hypothesis here is that both accelerating and self-limiting physical evolution derive from a common physically meaningful mechanism which may underlie a broad family of power-law kinetic behaviors.

**Figure 1:**
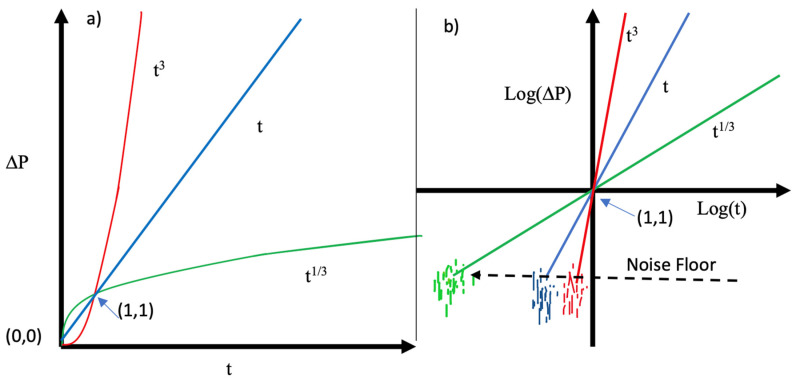
Panel (**a**) shows the evolution of an observable parameter ΔP on linear axes for n=3 (accelerating or cooperative evolution), n=1 (independent evolution), and n=1/3 (self-limiting or retarding evolution). These curves collectively originate from the same physical initial condition, ΔP=0 at t=0. Panel (**b**) shows the same relationships as (**a**) on log-log axes, where the kinetic exponent is represented by the slope.

To illustrate this concept, Figure 1 presents three classes of power-law evolution corresponding to accelerating, independent, and self-limiting behavior. The noisy regions in panel (b) is a log representation of the experimental noise floor, illustrating the practical difficulty of observing behavior near the origin. Early-time measurements are seen to be limited by the log of zero-centered noise and finite resolution; the underlying physical process is assumed to evolve continuously from the initial state. The three regimes depicted here demonstrate that accelerating $\left(n>1\right)$, independent $\left(n=1\right),$ and self-limiting 0<n<1 behavior may be represented within the common power-law form. 

In addition to the power-law dependence on time, many degradation processes exhibit a power-law dependence on applied stress. For a generic stress variable Ψ, the stress and temperature dependence of the rate coefficient is commonly represented as

(3) $$A\left(\Psi ,T\right)\propto {\left(\frac{\Psi }{\Psi_{0}}\right)}^{\gamma }\exp\left(-\frac{E_{A}}{kT}\right)$$

where Ψ may represent voltage, electric field, current density, mechanical strain, vibration amplitude, radiation flux, or chemical concentration, $\Psi_{0}$ is a characteristic scaling parameter, and $E_{A}$ is the apparent activation energy. The exponent γ describes the sensitivity of the degradation rate to the applied stress. While Equation (3) is commonly employed as an empirical acceleration relationship, its physical origin is often not explicitly addressed. As shown in Section 3, the stress acceleration term may be interpreted thermodynamically through a stress-dependent entropy contribution to the Gibbs free energy of activation. This interpretation provides a unified framework in which both the stress exponent γ and the kinetic exponent n arise from the statistical accessibility and correlation of microscopic states within the evolving system.

### 2.1. Three Classes of Kinetic Evolution

Figure 1 illustrates three representative forms of power-law evolution. The first corresponds to accelerating behavior characterized by n>1, where the probability of future evolution increases as the system evolves. The second corresponds to linear behavior with n=1, representing independent evolution in which the probability of future change remains constant. The third corresponds to self-limiting behavior characterized by 0<n<1, where the probability of future evolution decreases as the system evolves.

The linear-time representation emphasizes the continuous evolution of the observable parameter beginning from the initial state. In contrast, the log-log representation highlights differences in slope that are commonly used to determine kinetic exponents. The figure also illustrates the practical limitations associated with early-time measurements, where noise and finite measurement resolution may obscure behavior near the origin. Such experimental limitations should not be confused with the onset of the physical process itself. Fatigue damage begins with the first cycle [10], trapping begins with the first occupied state, and degradation begins with the first measurable perturbation even when the earliest stages cannot be directly observed.

The important observation here is that all three classes of behavior may be represented by the same mathematical form despite exhibiting dramatically different macroscopic evolution. The existence of these distinct regimes motivates the search for a common physical description capable of explaining; accelerating, independent, and self-limiting kinetics within a unified framework.

### 2.2. Cooperative Evolution: Accelerating Kinetics

One of the earliest and most influential examples of power-law behavior appears in the study of mechanical fatigue. Coffin observed that the number of cycles to failure is related to plastic strain through a power-law relationship [10], establishing a framework that remains widely used to this day in fatigue analysis. Subsequent developments, including the Coffin-Manson relationship, demonstrated the broad applicability of power-law descriptions to cumulative damage processes [11].

From a physical perspective, fatigue provides an example of cooperative evolution. The formation of microscopic defects, voids, or cracks alters the local stress distribution and increases the likelihood of further damage. In this sense, prior evolution promotes subsequent evolution. As damage accumulates, the probability of future degradation increases, producing accelerating behavior that is qualitatively consistent with n>1.

Similar accelerating behavior has been observed in a variety of cumulative damage processes, including solder-joint fatigue, crack propagation, dielectric breakdown, and other cooperative failure mechanisms [15,16]. Although the microscopic physics differs among these systems, the common feature is that the evolving state of the system increases the likelihood of subsequent evolution.

The existence of accelerating kinetics suggests that the system retains information regarding its prior evolution. This acts as a memory effect within the material, so that the following microstate is affected by the present state of accumulated microstates. Rather than behaving as a collection of independent events, the future state depends on the accumulated history of the system. This observation provides the first indication that interaction and correlation effects may play a fundamental role in determining kinetic behavior.

### 2.3. Independent Evolution: Classical Activated Kinetics

The simplest kinetic behavior occurs when individual transition events are statistically independent of one another. In such systems, the probability of a future transition is unaffected by the prior history of the system, and the evolution proceeds without either cooperative enhancement or self-limiting suppression. This is the classical Arrhenius description of activated processes which provides the most familiar example of this behavior [1].

Reaction rates are determined by an activation barrier and a thermal population factor, leading to kinetic behavior governed by a constant probability of activation. The subsequent development of transition-state theory by Eyring generalized this description through the Gibbs free energy of activation while preserving the concept of statistically independent transition events [3,4].

From the perspective of the power-law representation introduced above, independent evolution corresponds to the limiting case of n=1. In this regime, the accumulated change in the observable parameter increases linearly with time because the probability of future evolution remains constant throughout the process. No memory of prior evolution is retained by the system, and each microscopic transition occurs independently of previous transitions.

This independent linear regime serves as an important reference to the time law because it separates accelerating and self-limiting behavior. As will be shown, the classical Arrhenius-Eyring description emerges as a special limiting case of zero correlation within the broader framework developed in this work.

### 2.4. Self-Limiting Evolution: Retarding Kinetics

A third class of behavior is observed in systems where the probability of future evolution decreases as the system evolves. In contrast to cooperative processes, the accumulation of prior change inhibits subsequent evolution, producing progressively slower kinetics. Such behavior is frequently observed in degradation, trapping, relaxation, and occupancy-limited processes.

Examples include bias temperature instability in advanced semiconductor technologies, charge trapping in dielectric and interface states, and a variety of degradation phenomena in silicon, SiC, and GaN devices [12,13,14,17]. In many of these systems, the accumulated degradation follows a sub-linear power law characterized by 0<n<1. Although the microstates and associated mechanisms remain the subject of continuing modern-day investigation, the common experimental observation is that the rate of evolution decreases as the observable parameter accumulates.

From a phenomenological perspective, self-limiting behavior suggests that the evolving state of the system modifies the accessibility of future states. Occupied trapping sites, local field redistribution, screening effects, relaxation processes, and other interaction mechanisms may progressively reduce the probability of subsequent transitions. The resulting kinetics exhibit a form of memory in which prior evolution suppresses future evolution.

The prevalence of sub-linear power-law behavior across a wide range of physical systems suggests that self-limiting evolution is not restricted to a single microscopic mechanism. Instead, it may reflect more general thermodynamic principles associated with evolving occupancy, disorder, and interactions.

The existence of all three regimes; accelerating, independent, and self-limiting evolution raises a fundamental question. Are these three kinetic regimes fundamentally different phenomena requiring separate descriptions, or do they represent different manifestations of a common underlying thermodynamic framework? The remainder of this work explores this hypothesis.

## 3. Thermodynamic Origin of Correlation Entropy

The historical development reviewed in Section 1 suggests that kinetic evolution should be described in terms of the Gibbs free energy rather than a fixed activation barrier alone. Following Eyring [3,4], the rate, R, of an activated process may be expressed as

(4) $$R=R_{0}exp\left(-\Delta G/kT\right)$$

where R_0_ is an initial rate prefactor, k is Boltzmann’s constant, T is the absolute temperature, and ΔG is the Gibbs free energy governing the transition.

The experimental observations discussed in Section 2 suggest that the probability of future evolution may depend upon the current state of the system. In cooperative systems, prior evolution increases the probability of subsequent evolution, while in self-limiting systems prior evolution suppresses future evolution. Such behavior implies that the accessibility of future microstates may itself evolve as the system changes.

From classical statistical mechanics, the entropy of a system is related to the number of accessible microstates through the Boltzmann relationship

(5) $$S=k\;ln\left(\Omega \right)$$

where Ω represents the total number of accessible microscopic configurations. The introduction of a correlation-dependent entropy term requires some justification. Alternative generalized entropy formulations have previously been proposed, including the non-extensive entropy of Tsallis [18] and the generalized information entropy of Rényi [19]. These approaches modify the mathematical form of the entropy measure itself to account for correlations, long-range interactions, or departures from conventional Boltzmann-Gibbs statistics.

The present work follows a different approach. Rather than modifying the fundamental statistical definition of entropy, we retain the classical Boltzmann relationship of Equation (5). We hypothesize instead that, in an evolving system, the accessible multiplicity may be represented as

$$\Omega =\Omega_{\Psi }\Omega_{corr}$$

where $\Omega_{\Psi }$ represents the stress-dependent multiplicity associated with microstates made accessible by an applied stress, *Ψ*, consistent with Equations (2) and (3), and $\Omega_{corr}$ represents an additional multiplicity associated with the evolving state of the system. This latter contribution may arise from interactions, occupancy effects, disorder, screening, relaxation, or other mechanisms through which prior evolution influences the accessibility of future states.

Substituting this expression into Equation (5) gives

$$S=kln\left(\Omega_{\Psi }\Omega_{corr}\right)$$

or

$$S=kln\left(\Omega_{\Psi }\right)+kln\left(\Omega_{corr}\right)$$

The first term corresponds to the entropy associated with stress-accessible microstates. The second term represents an additional entropy contribution associated with the evolving correlations within the system. Motivated by the experimental observations of cooperative and self-limiting kinetics discussed in Section 2, we propose that this contribution scales with the accumulated number of participating microstates, *N*, according to

(6) $$\Delta S_{corr}=k\;\chi \;ln\left(N\right)$$

where χ is a dimensionless Correlation Constant that characterizes the degree to which prior microstate evolution influences subsequent evolution. Positive values of χ correspond to cooperative behavior in which prior evolution increases the probability of future evolution. Negative values correspond to self-limiting behavior in which prior evolution suppresses subsequent evolution. The limiting case χ = 0 corresponds to statistically independent evolution.

Equation (6) is introduced as a phenomenological extension of the classical entropy expression motivated by correlated kinetic evolution. The physical hypothesis underlying this formulation is that the evolving state of a system may alter the accessibility of future microstates through interaction, occupancy, disorder, screening, relaxation, or other correlation mechanisms. If such effects are present, they contribute an additional entropy term that modifies the Gibbs free energy and therefore the kinetic evolution itself.

The statistical definition of entropy by Boltzmann provides a natural connection between microscopic states and macroscopic thermodynamic behavior. To account for externally applied stress, we introduce a stress-dependent entropy contribution

(7) $$\Delta S_{\Psi }=k\gamma ln(\Psi /\Psi_{0})$$

then the total entropy contribution becomes

(8) $$\Delta S=\Delta S_{corr}+\Delta S_{\Psi }.$$

Substituting Equations (6) and (7),

(9) $$\Delta S=k\chi ln(N)+k\gamma ln(\Psi /\Psi_{0})$$

then

(10) ΔG=ΔH−TΔS

and

(11) $$\Delta G=\Delta H-kT\chi ln(N)-kT\gamma ln(\Psi /\Psi_{0})$$

where ΔH represents the intrinsic activation enthalpy of the unstressed system. The corresponding kinetic rate equation becomes

(12) $$dN/dt=\nu_{0}exp\left(-\Delta G/kT\right)$$

Substituting Equation (11) into Equation (12) yields

(13) $$dN/dt=\nu_{0}exp\left(-\Delta H/kT\right)\;N^{\chi }{\left(\Psi /\Psi_{0}\right)}^{\gamma }$$

Then, defining the thermal rate constant gives

(14) $$k_{r}=\nu_{0}exp\left(-\Delta H/kT\right)$$

which results in the rate function,

(15) $$dN/dt=k_{r}N^{\chi }{\left(\Psi /\Psi_{0}\right)}^{\gamma }$$

and then separating variables,

(16) $$N^{\left(-\chi \right)}dN=k_{r}{\left(\Psi /\Psi_{0}\right)}^{\gamma }dt$$

Integration then yields

(17) $$N^{\left(1-\chi \right)}/\left(1-\chi \right)=k_{r}{\left(\Psi /\Psi_{0}\right)}^{\gamma }t$$

and therefore

(18) $$N\left(t\right)={\left[\left(1-\chi \right)k_{r}{\left(\Psi /\Psi_{0}\right)}^{\gamma }t\right]}^{\left(1/\left(1-\chi \right)\right)}$$

The resulting kinetics exhibit the power-law form

(19) $$N\left(t\right)\propto t^{\left(1/\left(1-\chi \right)\right)}$$

Since the empirical power-law relationship is

$$N\left(t\right)\propto t^{n}$$

then it follows that

(20) $$n=1/\left(1-\chi \right)=1/m$$

then we can define

(21) χ=1−m.

Hence, we see that the physical interpretation of χ determines the nature of the resulting kinetics:

1. For χ = 0, the correlation entropy contribution vanishes, and the system reduces to classical Arrhenius-Eyring behavior with a constant activation barrier and linear evolution (m = 1, n = 1).
2. For χ < 0, the accumulation of microstates increases the effective Gibbs free-energy barrier through the correlation term. Future transitions become progressively less probable, producing self-limiting or retarding kinetics characterized by m > 1 and n < 1. This behavior is commonly observed in charge trapping, relaxation, and bias-temperature-instability phenomena.
3. For χ > 0, the accumulation of microstates lowers the effective barrier and increases the probability of future transitions. Evolution accelerates with time, producing cooperative kinetics characterized by m < 1 and n > 1. Examples include fatigue, crack propagation, dielectric breakdown, and other positive-feedback degradation processes.

Thus, independent, self-limiting, and cooperative evolution emerge naturally from a common thermodynamic framework. Within this interpretation, the kinetic exponent reflects the degree of correlation among microscopic states, while the stress exponent γ describes how external driving forces modify the accessibility of those states.

## 4. Physical Interpretation of the Correlation Constant

The derivation of Section 3 demonstrates that the Correlation Constant, χ, modifies the effective Gibbs free energy according to Equation (11). This relationship provides a direct thermodynamic interpretation of χ and establishes the physical origin of the observed power-law kinetics. Rather than viewing the kinetic exponent as an empirical fitting parameter, the present framework now interprets the exponent as a measurable consequence of a correlation-dependent modification to the free-energy landscape.

**Figure 2:**
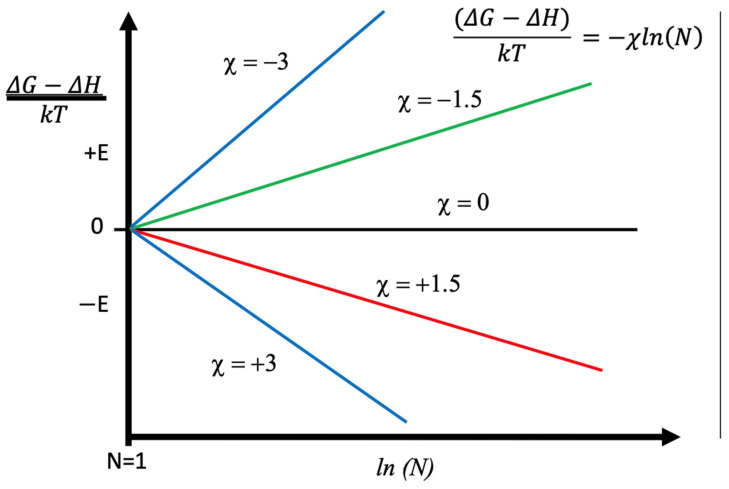
Normalized effective free-energy shift as a function of accumulated microstate evolution *ln*N. From $\left(\Delta G-\Delta H\right)/kT=-\chi lnN$, where χ determines the sign and magnitude of the evolving free-energy change. Positive χ lowers the effective barrier with increasing *ln*N, producing cooperative evolution. Negative χ raises the effective barrier, producing self-limiting behavior. The case χ=0 recovers the Arrhenius-Eyring limit with a constant barrier.

Within the present framework, an evolving system begins from an intrinsic activation barrier, ΔH, which is also known as the activation energy, $E_{a}$. The progression of ΔG then follows from the initial number of accumulated microstates over time, assuming at zero time, N=1, and ln(N)=0. As evolution proceeds further in time, the correlation energy alters the accessibility of future states. The sign and magnitude of the Correlation Constant, χ, determine whether the effective barrier increases, decreases, or remains unchanged. Figure 2 illustrates the normalized free-energy shift expressed by rearranging Equation (11) with zero stress ($\Psi /\Psi_{0}=1$), where we plot:

(22) $$\frac{\Delta G-\Delta H}{kT}=-\chi \ln\left(N\right)$$

for representative values of the Correlation Constant, χ. Positive values of χ produce a decreasing effective barrier with increasing system evolution, while negative values produce an increasing barrier. The special case, χ=0, corresponds to the classical Arrhenius-Eyring description in which the activation barrier remains constant throughout the evolution process. For convenience, define the correlation energy as

(23) $$E_{corr}=kT\chi ln(N)$$

corresponding to the χ-dependent contribution to Equation (11).

Figure 2 illustrates the χ-dependent free-energy contribution to Δ*G*. The horizontal axis represents the accumulated number of microscopic states participating in the evolution process through the variable ln(N), while the vertical axis represents the corresponding normalized free-energy shift. As degradation, relaxation, transport, or structural evolution proceeds, each microscopic transition contributes to the accumulated state count. Depending on the physical system, these microstates may correspond to trapped charges, defect configurations, dislocation arrangements, local strain states, diffusion events, or other microscopic realizations of the evolving system. The logarithmic dependence on correlation entropy in Equation (11) follows directly from the statistical definition of entropy as we see in Equation (6), while χ determines the degree to which the evolving system increases or decreases the accessibility of future states.

### 4.1. Positive Correlation, χ>0

For positive values of χ, the correlation energy is positive and increases with increasing system evolution, $E_{corr}>0.$ Consequently, the effective Gibbs free energy decreases as the system evolves. Existing damage or evolution therefore promotes accumulation of defects by making subsequent transitions increasingly accessible. This behavior produces cooperative kinetics and accelerating evolution characterized by (n>1). Examples include fatigue damage, crack propagation, dielectric breakdown, and other cumulative failure processes in which prior evolution increases the probability of subsequent evolution. In thermodynamic terms, the evolving system progressively lowers its effective barrier, leading to increasingly rapid change.

### 4.2. Zero Correlation, χ=0

When the Correlation Constant is zero, the correlation entropy contribution vanishes, that is, $E_{corr}=0$. Then, the effective free energy remains constant, ΔG=ΔH, and the probability of future transitions is independent of prior microstates. This regime corresponds directly to the classical Arrhenius-Eyring framework and produces linear kinetics with n=1. This zero-correlation condition therefore serves as the boundary separating cooperative and self-limiting evolution and represents the special case in which microscopic transitions occur independently of the evolving system state.

### 4.3. Negative Correlation, χ<0

For negative values of the Correlation Constant, χ, the correlation energy is negative. Then, $E_{corr}<0,$ and the effective Gibbs free energy increases with increasing evolution. In this regime, prior accumulation inhibits future evolution by progressively reducing the accessibility of available states. The probability of subsequent transitions decreases as the system evolves, producing self-limiting behavior characterized by 0<n<1. Examples include charge trapping, bias-temperature instability, occupancy-limited processes, and relaxation phenomena in which occupied states, local field redistribution, screening effects, or configurational constraints reduce the likelihood of further evolution. The resulting kinetics become progressively slower because the evolving system raises its own effective free-energy barrier.

### 4.4. Unified Interpretation

Figure 2 demonstrates that cooperative, independent, and self-limiting evolution may all be understood as consequences of a single thermodynamic relationship governed by the Correlation Constant. Positive correlation lowers the effective free-energy barrier, negative correlation raises the barrier, and zero correlation leaves the barrier unchanged.

(24) $$\frac{\partial }{\partial lnN}\left(\frac{\Delta G-\Delta H}{kT}\right)=-\chi .$$

Therefore, the Correlation Constant, χ, may be interpreted as the slope of the normalized free-energy landscape. Within this interpretation, χ becomes a fundamental thermodynamic quantity, while the power-law exponent represents its experimentally observable manifestation. The diversity of observed kinetic behaviors therefore emerges from differences in the evolution of the free-energy landscape rather than from unrelated empirical laws.

## 5. Implications of Correlated Kinetics

The principal outcome of this work is that we can view the power-law time exponent not merely as an empirical fitting parameter, but rather as an intrinsic property of accumulation, degradation and structural evolution under stress. Through the Correlation Constant, χ (defined as χ=1−m where m=1/n), the observed power-law relationship of Equation (2), $\Delta P\left(\Psi ,T,t\right)=A\left(\Psi ,T\right)t^{n\left(\Psi ,T\right)}$, has a physical meaning. The time exponent, *n,* then serves as a measurable indicator of the underlying kinetic correlations governing system evolution. The kinetic exponent n(Ψ,T), the exponent parameter m=1/n, the Correlation Constant χ, and the correlation energy from Equation (23) therefore represent complementary descriptions of the same underlying thermodynamic interaction. Accelerating, independent, and self-limiting kinetic behavior emerge as different manifestations of a common thermodynamic framework rather than unrelated empirical observations.

Experimental observations frequently indicate that the kinetic exponent varies with both stress and temperature. Within the present framework, this behavior suggests that the Correlation Constant is itself a function of stress, $\chi =\chi \left(\Psi \right)$, where stress may represent voltage, electric field, current density, mechanical strain, vibration amplitude, radiation flux, chemical concentration, or other external driving forces. The correlation energy, $E_{corr}=kT\chi ln(N)$, is proportional to temperature and therefore provides a measurable link between operating conditions and the underlying degree of interaction within the evolving system where the correlation energy depends directly on *T*.

The present interpretation provides a natural connection between a broad range of experimental observations. Classical Arrhenius-Eyring kinetics correspond to the special case of zero correlation. Fatigue and solder-joint degradation described by the Coffin-Manson framework, however, represent examples of positive correlation, where existing damage promotes future damage. Conversely, BTI, charge trapping, and related degradation phenomena in Si, SiC, and GaN technologies represent examples of negative correlation, where accumulated evolution progressively inhibits further evolution. Within the present framework, these apparently distinct phenomena are unified through the Correlation Constant and its associated correlation entropy and energy.

One immediate consequence of this interpretation is that the kinetic exponent contains physical information that is generally absent from conventional Arrhenius analysis. Traditional approaches often focus on activation energy as the defining characteristic of a degradation mechanism. The present formulation suggests that the kinetic exponent, $n\left(\Psi ,T\right),$ provides complementary information regarding the degree of interaction within the evolving system. A value of n = 1 corresponds to independent evolution and recovers the classical Arrhenius-Eyring framework. Values of n > 1 indicate positive correlation, while values of 0 < n < 1 indicate negative correlation and self-limiting evolution.

The present framework also suggests a new perspective for reliability engineering and materials science. Rather than treating the power-law exponent solely as a fitting parameter, the exponent may be interpreted as a measurable thermodynamic quantity reflecting the degree of interaction within the evolving system. Changes in stress, temperature, material structure, or processing conditions that alter the exponent may therefore provide direct information regarding changes in the underlying correlation mechanisms.

More broadly, the results suggest that power-law kinetics may not simply represent convenient empirical approximations, but instead reflect a fundamental connection between thermodynamics, entropy, correlation, and system evolution. Within this interpretation, the classical Arrhenius description corresponds to the special case of zero correlation; while accelerating and self-limiting behavior emerge naturally from positive and negative correlation, respectively. The observed diversity of kinetic behavior may therefore arise from a single thermodynamic principle governed by the correlation-dependent contribution to the Gibbs free energy.

The present framework is falsifiable. If power-law kinetics are observed while the extracted value of χ fails to consistently predict the observed evolution, or if systems exhibiting identical values of χ demonstrate fundamentally different kinetic behavior, the proposed interpretation would require revision. Likewise, if independent measurements of microscopic correlations are found to be uncorrelated with the extracted χ values, the hypothesis would be weakened. Future work should therefore focus on establishing experimental relationships between χ and independently measurable physical quantities.

## 6. Activation Energy, Correlation, and Recoverability

The correlated kinetic framework developed in this work suggests that activation energy and kinetic exponent should not be viewed as independent quantities. Instead, both emerge from a common thermodynamic description through the correlation-dependent contribution to the Gibbs free energy. While activation energy characterizes the intrinsic barrier governing an elementary process, the Correlation Constant characterizes how the evolving state of the system modifies the accessibility of future states.

Experimental studies of BTI and related degradation phenomena have repeatedly demonstrated that the power-law exponent varies with stress and temperature [12]. Similar observations have been interpreted through disorder, dispersion, and distributed activation barriers [20]. Within the present framework, these observations suggest that the Correlation Constant itself is a function of stress and temperature, $\chi =\chi \left(\Psi \right),$ and that the observed exponent contains physical information regarding the evolving thermodynamic state of the system.

The dependence of the Correlation Constant on stress may be particularly significant. In many physical systems, stress not only increases the rate of evolution but also alters the interactions among the evolving states themselves. Increased electric field, voltage, current density, mechanical strain, vibration amplitude, radiation flux, or chemical driving force may modify occupancy, local fields, defect populations, relaxation processes, and configurational accessibility. Consequently, stress may influence not only the intrinsic activation barrier but also the degree of correlation within the evolving system. Within the present framework, variations in the observed power-law exponent with operating conditions may therefore be interpreted as changes in the Correlation Constant, suggesting that the exponent contains information regarding how the applied stress modifies the evolving free-energy landscape.

Several independent studies have reported a systematic temperature dependence of the power-law exponent in BTI degradation, including the work of Huard et al. [12], Alam and Mahapatra [21], Yang et al. [22], and Kaczer et al. [23]. In each case, the experimental data may be approximated by a relation of the form

(25) $$m\approx 1+\frac{\alpha }{kT}\;,$$

where α is a characteristic energy scale on the order of 0.05–0.10 eV. This range is consistent with previous observations suggesting that the intrinsic energy scale governing BTI degradation may be substantially smaller than conventionally extracted activation energies [24].

(26) $$\chi =1-m\approx -\frac{\alpha }{kT}\;,$$

and therefore, the correlation energy,

(27) $$E_{corr}=kT\;\chi \;\ln N\approx -\alpha \ln N.$$

With this interpretation, the observed temperature dependence of the exponent arises naturally from an approximately temperature-independent correlation-energy scale. The power-law exponent is therefore not the fundamental quantity, but rather an observable consequence of the Correlation Constant and its associated correlation energy. This observation further supports the interpretation of the Correlation Constant as a physically meaningful thermodynamic parameter rather than a simple mathematical transformation of the power-law exponent.

The stress term, Ψ, may be viewed as modifying the number of accessible configurations available to the evolving system. Increased stress increases the accessibility of microstates and therefore lowers the effective free-energy barrier through the stress-dependent entropy contribution. In this interpretation, γ quantifies the sensitivity of the accessible-state population to applied stress.

The present interpretation does not require a unique microscopic origin for the Correlation Constant. Disorder, occupancy effects, trapping, screening, local relaxation, configurational evolution, and distributed activation barriers may all contribute to the correlation-dependent entropy term. In this sense, previously proposed explanations based on dispersion or disorder may be viewed as contributors to the Correlation Constant rather than alternative explanations for power-law behavior.

One consequence of this interpretation is that the apparent activation energy obtained from conventional analysis may substantially exceed the intrinsic microscopic barrier. Previous analyses have demonstrated a strong coupling between activation energy and power-law exponent in BTI degradation [24]. For negatively correlated systems, where m>1, the intrinsic barrier may therefore be significantly smaller than the experimentally observed value.

A reduced intrinsic barrier carries important physical implications. If the elementary process is governed by a relatively small activation enthalpy, the evolving system may remain close to thermodynamic equilibrium and may access a broad range of nearby configurational states. The observed degradation need not correspond to irreversible structural damage. Instead, it may reflect changes in occupancy, local configuration, interaction energy, or configurational accessibility.

Such systems are expected to exhibit substantial recovery when the external stress is removed or reversed. Recovery, relaxation, annealing, and self-healing may therefore be viewed as natural consequences of a system whose evolution is governed by a low intrinsic barrier combined with a negative Correlation Constant. This interpretation may help explain the substantial recovery frequently observed in BTI and related trapping phenomena, where degradation appears to be associated with evolving occupancy and interaction states rather than permanent defect generation.

More broadly, the combined consideration of activation energy and Correlation Constant provides a more complete thermodynamic description of degradation and structural evolution under stress. Activation energy, defined here as the enthalpy, ΔH, characterizes the intrinsic barrier to evolution, while the Correlation Constant characterizes how prior evolution modifies future evolution. Together, these quantities provide complementary information regarding stability, recoverability, and long-term system behavior.

Experimental studies of fatigue-damaged steels have similarly demonstrated that substantial recovery can be achieved through annealing when the dominant damage remains at the microstructural level, whereas the formation of microcracks leads to irreversible degradation. Such observations are consistent with the present interpretation that low-energy correlated states may be recoverable, while permanent structural damage introduces irreversible changes to the evolving free-energy landscape [25].

The characteristic correlation-energies may be on the order of only 0.05–0.10 eV, only a few *kT* at most. This suggests that a substantial fraction of experimentally observed degradation may correspond to low-energy configurational evolution rather than irreversible structural damage. Such systems would therefore be expected to exhibit significant recovery when stress is removed, and sufficient thermal energy is available to restore access to previously relaxed configurations. Other studies in reliability physics have identified both recoverable and irreversible degradation components associated with evolving trapped-state populations and occupancy-dependent kinetics, although the thermodynamic interpretation of the resulting kinetic exponents remains unresolved [26].

This perspective suggests that future studies should treat the power-law exponent not merely as a fitting parameter, but as a measurable physical quantity containing information regarding the underlying kinetic interactions. The simultaneous determination of activation energy and Correlation Constant may therefore provide a powerful framework for distinguishing among competing physical mechanisms and for identifying pathways toward improved reliability, enhanced recoverability, and ultimately the control of degradation processes.

## 7. Conclusions

The historical development of activated processes has progressed from the empirical Arrhenius description of reaction rates, through the thermodynamic formulation of Eyring, to modern recognition of the importance of disorder, occupancy, and interaction effects in complex systems. While these developments have greatly expanded our understanding of kinetic behavior, power-law evolution remains one of the most widely observed yet least understood features of physical systems undergoing degradation, relaxation, transport, and structural evolution.

In this hypothesis, a thermodynamic framework for correlated kinetics has been developed by introducing a correlation-dependent entropy contribution to the Gibbs free energy. Motivated by the statistical definition of entropy and by the role of occupancy and interaction effects in evolving systems, a Correlation Constant, χ, was introduced to characterize the influence of prior evolution on the accessibility of future states. This approach leads naturally to a correlation energy of the form $E_{corr}=kT\chi \ln N,$ which modifies the effective free-energy landscape as the system evolves.

The resulting formulation provides a direct connection between thermodynamics and the widely observed power-law relation $\Delta P\left(\Psi ,T,t\right)=A\left(\Psi ,T\right)t^{n\left(\Psi ,T\right)}.$ Rather than treating the power-law exponent only as an empirical fitting parameter, this work hypothesizes that the exponent is a measurable indicator of correlation within the evolving system. Positive values of the Correlation Constant produce cooperative evolution, negative values produce self-limiting evolution, and zero correlation recovers the classical Arrhenius-Eyring description as a special limiting case.

This interpretation provides a common framework for understanding; accelerating, independent, and retarding kinetic behavior. Fatigue and cumulative damage processes may be viewed as manifestations of positive correlation, while trapping, relaxation, and bias-temperature-instability phenomena represent examples of negative correlation. Although the microscopic origins of these behaviors may differ, their kinetic evolution can be described within a common thermodynamic perspective through a measurable correlation-dependent contribution to the Gibbs free energy.

Experimental observations indicate stress- and temperature-dependent exponents, which imply that the Correlation Constant itself may depend on applied stress $\chi =\chi \left(\Psi \right).$ Consequently, the exponent contains physical information regarding the evolving state of the system and the underlying interaction mechanisms. The resulting correlation energy generally depends on temperature through kT χ, although experimental observations suggest that in some systems the product may approach a temperature-independent energy scale. Thus, measured apparent activation energies may include contributions arising from correlation effects and that the intrinsic microscopic enthalpy ($E_{A}$) may be substantially smaller than conventionally inferred.

More broadly, the results suggest that power-law behavior may not simply represent a convenient empirical approximation, but rather a thermodynamic signature of correlated evolution. Within this interpretation, the diversity of observed kinetic behavior emerges from a common free-energy framework in which entropy, occupancy, disorder, and interaction effects modify the probability of future transitions. The classical Arrhenius description is therefore but a special case of a more general thermodynamic continuum spanning cooperative, independent, and self-limiting evolution. The present work should therefore be viewed as a thermodynamic hypothesis intended to stimulate further experimental and theoretical investigation of the relationship between entropy, correlation, and kinetic evolution.

Future work should focus on establishing the dependence of the Correlation Constant on stress, temperature, and material structure, as well as identifying the microscopic mechanisms that contribute to correlation in specific physical systems. If validated experimentally, the Correlation Constant may provide a useful thermodynamic quantity for interpreting degradation, recoverability, and long-term evolution across a broad range of scientific and engineering disciplines.

## 8. Key Findings

- A correlation-dependent entropy term naturally produces power-law kinetics.
- The Correlation Constant, χ, may depend on stress and temperature.
- Positive correlation produces accelerating evolution.
- Negative correlation produces self-limiting evolution.
- Arrhenius-Eyring kinetics correspond to the special case χ=0.
- Activation energy and χ contain complementary physical information.

## Data Availability

There is no new data presented here, only a Hypothesis.
